# Supplementary material for: CUG initiation and frameshifting enable production of dipeptide repeat proteins from ALS/FTD C9ORF72 transcripts
Source: Nat Commun. 2018 Jan 11;9:152. doi: 10.1038/s41467-017-02643-5 (PMC5764992; doi:10.1038/s41467-017-02643-5)
Supplement: Supplementary file 1 — Supplementary Information [file 41467_2017_2643_MOESM1_ESM.pdf]

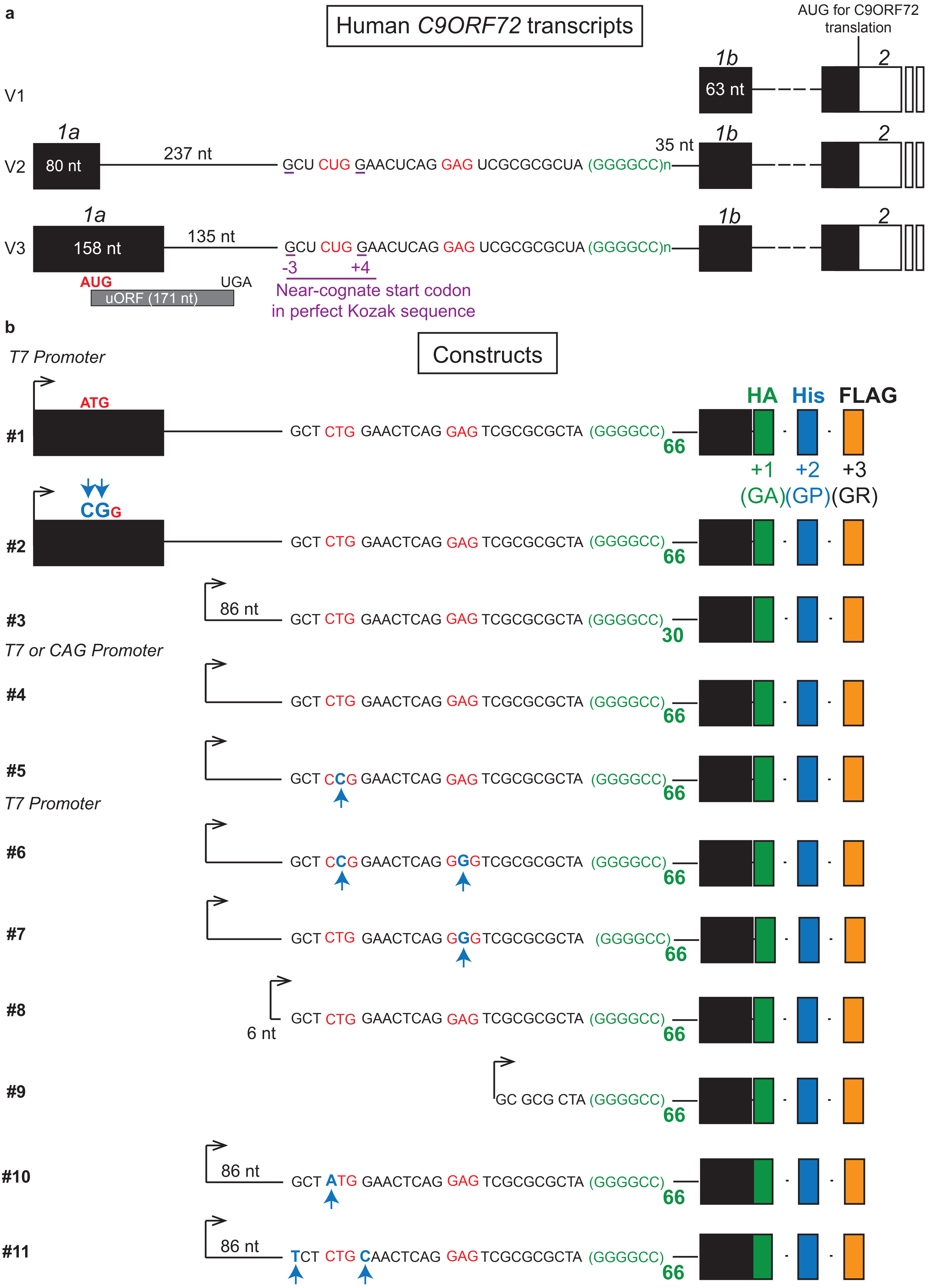
a
Human C9ORF72 transcripts
AUG for C9ORF72 translation
V1
1b
63 nt
2
V2
1a
80 nt
237 nt
GCU CUG GAACUCAG GAG UCGCGCGCUA (GGGGCC)n
35 nt
1b
2
V3
1a
158 nt
135 nt
GCU CUG GAACUCAG GAG UCGCGCGCUA (GGGGCC)n
1b
2
-3 +4
Near-cognate start codon in perfect Kozak sequence
AUG
UGA
uORF (171 nt)
b
Constructs
T7 Promoter
#1
ATG
GCT CTG GAACTCAG GAG TCGCGCGCTA (GGGGCC) 66
HA His FLAG
+1 +2 +3
(GA)(GP)(GR)
#2
CGG
GCT CTG GAACTCAG GAG TCGCGCGCTA (GGGGCC) 66
#3
86 nt
GCT CTG GAACTCAG GAG TCGCGCGCTA (GGGGCC) 30
T7 or CAG Promoter
#4
GCT CTG GAACTCAG GAG TCGCGCGCTA (GGGGCC) 66
#5
GCT CCG GAACTCAG GAG TCGCGCGCTA (GGGGCC) 66
T7 Promoter
#6
GCT CCG GAACTCAG GGG TCGCGCGCTA (GGGGCC) 66
#7
GCT CTG GAACTCAG GGG TCGCGCGCTA (GGGGCC) 66
#8
6 nt
GCT CTG GAACTCAG GAG TCGCGCGCTA (GGGGCC) 66
#9
GC GCG CTA (GGGGCC) 66
#10
86 nt
GCT ATG GAACTCAG GAG TCGCGCGCTA (GGGGCC) 66
#11
86 nt
TCT CTG CAACTCAG GAG TCGCGCGCTA (GGGGCC) 66

**Supplementary Figure 1. Schematic of *C9ORF72* constructs with ($G_4C_2$) repeats and mutations introduced in the 5' flanking sequence.**

**(a)** Schemes of human *C9ORF72* RNA isoforms with the $G_4C_2$ repeat located in the first intron of the gene. Isoform V1 starts at a downstream first exon (exon 1b) and does not contain the $G_4C_2$ repeat. Transcripts V2 and V3 start at exon 1a and contain the $G_4C_2$ repeat. Exons 1a, 1b, 2 (black boxes) and intron 1 (black line interrupted by nucleotides sequence) are presented with the location of the $G_4C_2$ repeat and the position of near-cognate start codons (CUG) and (GAG) that might serve as alternative start site for RAN translation. The potential CUG start codon is located in the +1 Glycine-Alanine frame and in a perfect Kozak sequence with G at -3 and G at +4 (labeled in purple). The grey box overlapping exon 1A and intron 1 represents a 171 nucleotides (nt) long upstream open reading frame (uORF) located 79 nucleotides upstream from the $G_4C_2$ repeat. **(b)** Schemes of various constructs with the full length sequence upstream of the $(G_4C_2)_{66}$ repeat (construct #1); the full length with AUG>CCG mutations in a codon that serves as start site for the uORF translation (construct #2); deletion mutation removing the uORF and keeping 113 nucleotides 5' to 30 repeats (construct #3) or 66 repeats (construct #4); C**T**G>C**C**G mutation (blue arrow) in a near-cognate start codon located 24 nucleotides upstream of the $(G_4C_2)_{66}$ repeat (construct #5); G**A**G>G**G**G mutation (blue arrow) in another putative start codon located 12 nucleotides upstream of the $(G_4C_2)_{66}$ repeats (construct #7); double C**T**G>C**C**G and G**A**G>G**G**G mutations (blue arrows) in the putative near-cognate start codons (construct #6); deletion removing 311 nucleotides in the 5' region of the gene and leaving 33 nucleotides upstream of the $(G_4C_2)_{66}$ repeat (construct #8); deletion removing the potential start codons CUG and GAG and leaving 8 nucleotides upstream of the $(G_4C_2)_{66}$ repeat (construct #9); CTG>ATG mutation (blue arrow) in the near-cognate start codon located 24 nucleotides upstream of the (G4C2)66 repeat(construct #10); double **G**CTCTG**G**>**T**CTCTG**C** mutations in the Kozak sequence surrounding the CUG codon (construct #11). All constructs have HA, His and FLAG tags in the +1 poly-GA, +2 poly-GP and +3 poly-GR frame, respectively. The full sequence of each construct is provided in **Supplementary Table 1**.

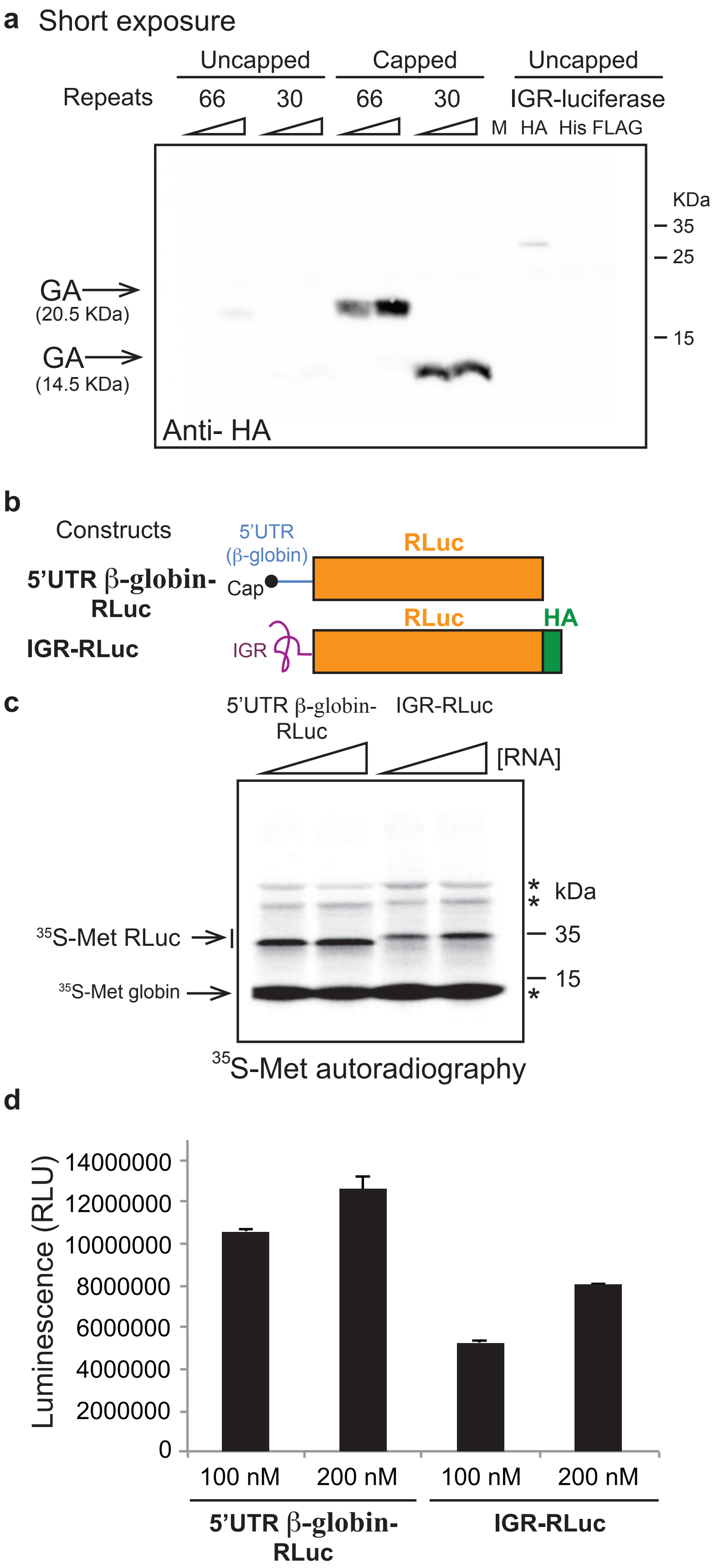


**Supplementary Figure 2: *In vitro* translation efficiency of IRES (IGR) comparatively to capped mRNA (5'UTR of β-globin) in RRL.**

**(a)** Short exposure of the immunoblot presented in Figure 1a **(b)** Schemes of Renilla Luciferase (RLuc) RNAs under the control of the intergenic region (IGR) of the capsid protein gene in dicistroviruses (IGR-RLuc) or capped 5' untranslated region of the β-globin gene (5'UTR-β-globin-RLuc). **(c)** Translation was performed in presence of [$^{35}$S]-methionine and IGR-RLuc and 5'UTR-β-globin-RLuc at 100 and 200 nM in RRL. Renilla luciferase products were detected by autoradiography. Asterisks indicate unspecific proteins translated in RRL such as β-globin. **(d)** Renilla luciferase activity were measured by Luminescence. Graphs represent mean ± SEM, n=3.

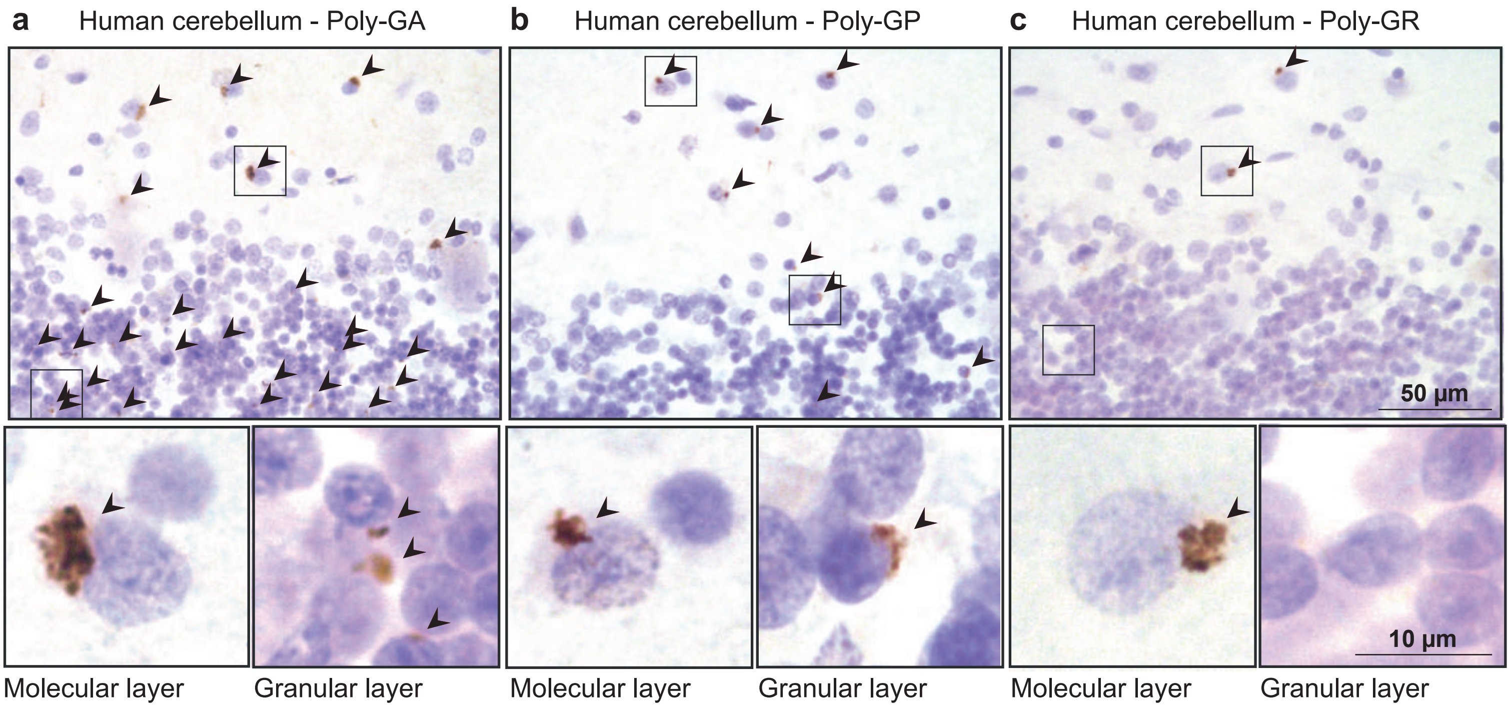


**Supplementary Figure 3: Burden of poly-GA, poly-GP and poly-GR aggregates in cerebellum from a *C9ORF72* ALS/FTD patient.**
Immunohistochemistry on post-mortem cerebellum from a *C9ORF72* ALS/FTD patient using **(a)** poly-GA, **(b)** poly-GP or **(c)** poly-GR antibodies. Magnifications of the molecular and granular layers are presented on the lower panels. Nuclei were stained with hematoxylin. Scale bars represent 50 µm and 10 µm in the upper and lower panels, respectively. Arrows highlight the poly-GA, poly-GP and poly-GR aggregates.

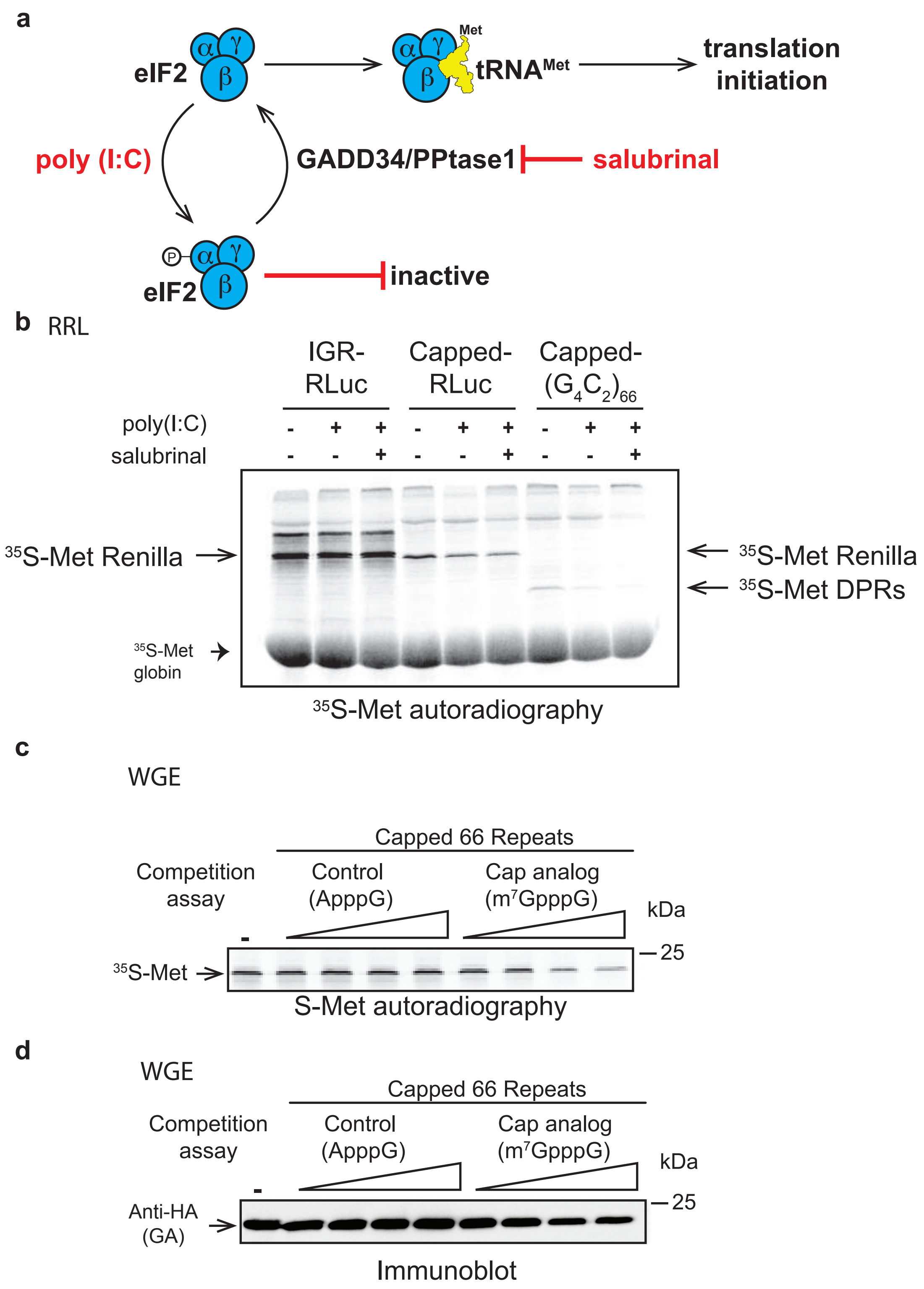


**Supplementary Figure 4: $G_4C_2$ RAN translation is cap-dependent and requires an active eIF2 complex with methionylated initiator tRNA**

**(a)** Scheme of the heterotrimer eIF2 assembly with the methionylated initiator $tRNA^{Met}$ in presence of salubrinal, specific inhibitor of GADD34/PPtase1 and poly (I:C), stimulator of eIF2α phosphorylation. **(b)** Translation in RRL was performed in presence of [$^{35}$S]-methionine, eIF2α inhibitors (15 µM Salubrinal, poly(I:C)) and 200 nM of IGR-Renilla luciferase (IGR-RLuc), capped-Renilla luciferase (Capped-RLuc) or capped 66 repeat transcript (construct #4, **Supplementary Fig. 1**). Translation products were detected by autoradiography. **(c and d)** Translation was performed in presence of [$^{35}$S]-methionine, capped $(G_4C_2)_{66}$ RNA #4 and an increased concentration of inactive cap (control, ApppG) or cap analog (competitor of the cap, m$^7$GpppG) in wheat germ extracts (WGE). [$^{35}$S]-methionine RAN translation products and DPR were detected by **(c)** autoradiography and **(d)** immunoblot with anti-HA (poly-GA) antibody.

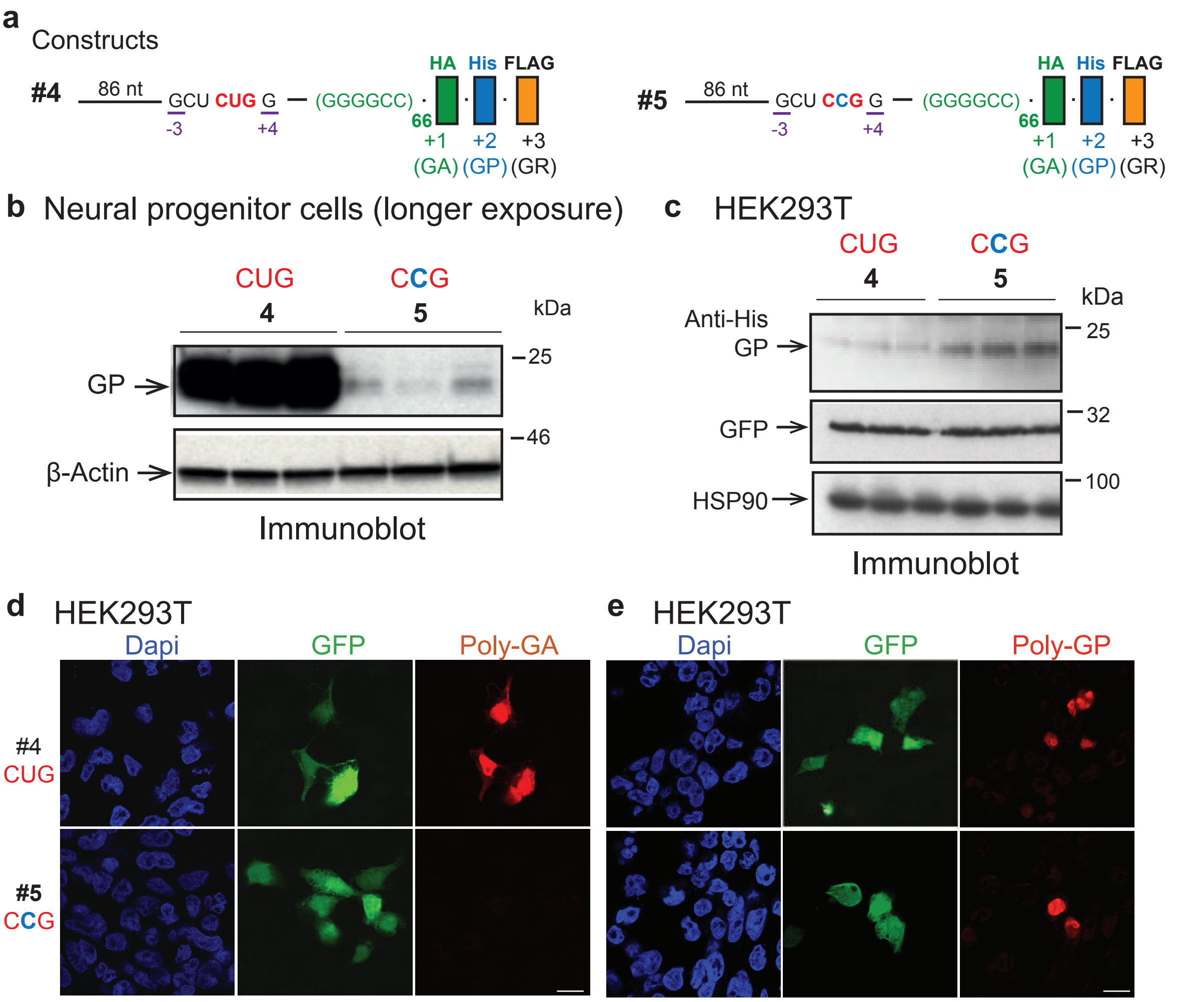


**Supplementary Figure 5: Mutation of the near-cognate start codon CUG alters RAN translation in human cells.**

**(a)** Schematic representation of constructs #4 or #5 containing either the near-cognate codon CUG or the mutant CUG>CCG 24 nucleotides upstream of $(G_4C_2)_{66}$ repeats, respectively. **(b)** Longer exposure of the poly-GP immunoblot presented in Figure 4b obtained in human neural progenitor cells transfected with constructs #4 or #5 and detected by an antibody against GP epitope. **(c)** Immunoblot of poly-GP proteins in HEK293T cells transfected with constructs #4 or #5, detected by an antibody against the HIS tag. GFP and HSP90 immunoblots are used as loading controls. Expression of poly-GA (red) **(d)** and poly-GP (red) **(e)** was determined by confocal immunofluorescence in HEK293T co-transfected with constructs #4 or #5 and GFP plasmid. Nuclei are visualized with Dapi. Scale bar, 20 µm

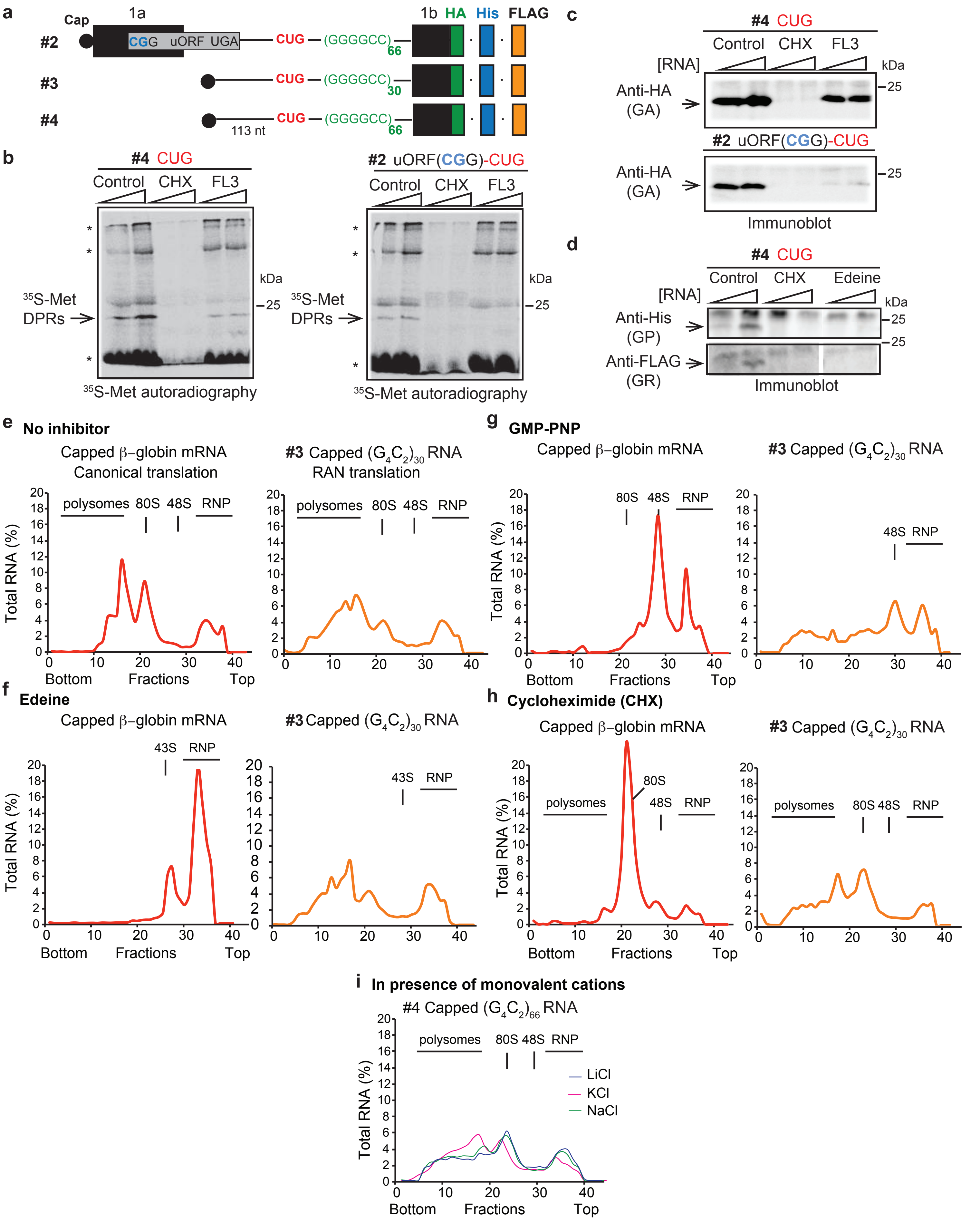
a
Cap
1a
1b HA His FLAG
#2 CGG uORF UGA CUG (GGGGCC)66
#3 CUG (GGGGCC)30
#4 113 nt CUG (GGGGCC)66
b
#4 CUG
Control CHX FL3
35S-Met DPRs
kDa
25
35S-Met autoradiography
#2 uORF(CGG)-CUG
Control CHX FL3
35S-Met DPRs
kDa
25
35S-Met autoradiography
c
#4 CUG
Control CHX FL3
[RNA]
kDa
Anti-HA (GA)
25
#2 uORF(CGG)-CUG
Anti-HA (GA)
25
Immunoblot
d
#4 CUG
Control CHX Edeine
[RNA]
kDa
Anti-His (GP)
25
Anti-FLAG (GR)
25
Immunoblot
e No inhibitor
Capped β–globin mRNA Canonical translation
polysomes 80S 48S RNP
#3 Capped (G4C2)30 RNA RAN translation
polysomes 80S 48S RNP
Total RNA (%)
Bottom Fractions Top
f Edeine
Capped β–globin mRNA
43S RNP
#3 Capped (G4C2)30 RNA
43S RNP
Total RNA (%)
Bottom Fractions Top
g GMP-PNP
Capped β–globin mRNA
80S 48S RNP
#3 Capped (G4C2)30 RNA
48S RNP
Total RNA (%)
Bottom Fractions Top
h Cycloheximide (CHX)
Capped β–globin mRNA
polysomes 80S 48S RNP
#3 Capped (G4C2)30 RNA
polysomes 80S 48S RNP
Total RNA (%)
Bottom Fractions Top
i In presence of monovalent cations
#4 Capped (G4C2)66 RNA
polysomes 80S 48S RNP
LiCl
KCl
NaCl
Total RNA (%)
Bottom Fractions Top

**Supplementary Figure 6: $G_4C_2$ transcripts undergo scanning-dependent RAN translation and associate with ribosomal subunits even in absence of translation.**

**(a)** Scheme of constructs #2, #3 and #4 used for translation and RNA profiling by polyribosome fractionation in RRL system. RNAs #2 and #4 are uORF mutants with a mutation of the AUG start codon or a deletion of 207 nucleotides removing the uORF. Both harbor 66 $G_4C_2$ repeats. Construct #3 is identical to #4 but with 30 repeats. **(b-d)** Translation inhibitors were used to investigate the mechanisms of RAN translation initiation. Translation inhibitions by cycloheximide (CHX, translation elongation inhibitor) or FL3 (eIF4 helicase inhibitor) were performed in presence of [$^{35}$S]-methionine and capped RNA #2 (long 5'end) or #4 (short 5' end). RAN translation products were detected **(b)** by autoradiography or (c) by immunoblot with an antibody against the HA tag (poly-GA). **(d)** Effects of cycloheximide and edeine (codon-anticodon interaction inhibitor) on poly-GP and poly-GR RAN translation identified by immunblots with antibodies against HIS (poly-GP) and FLAG (poly-GR). **(e-g)** RNA profiling by polyribosome fractionation were performed with radioactive capped $(G_4C_2)_{30}$ RNA (#3) and with radioactive capped β-globin mRNA **(e)** without inhibitor, **(f)** in presence of edeine, **(g)** GMP-PNP (inhibiting the assembly of 60S ribosomal subunit to form an 80S functional ribosome) or **(h)** CHX. **(i)** RNA profiling with capped $(G_4C_2)_{66}$ RNA (#4) in presence of different monovalent cations Li+, K+, Na+ at 50 mM.

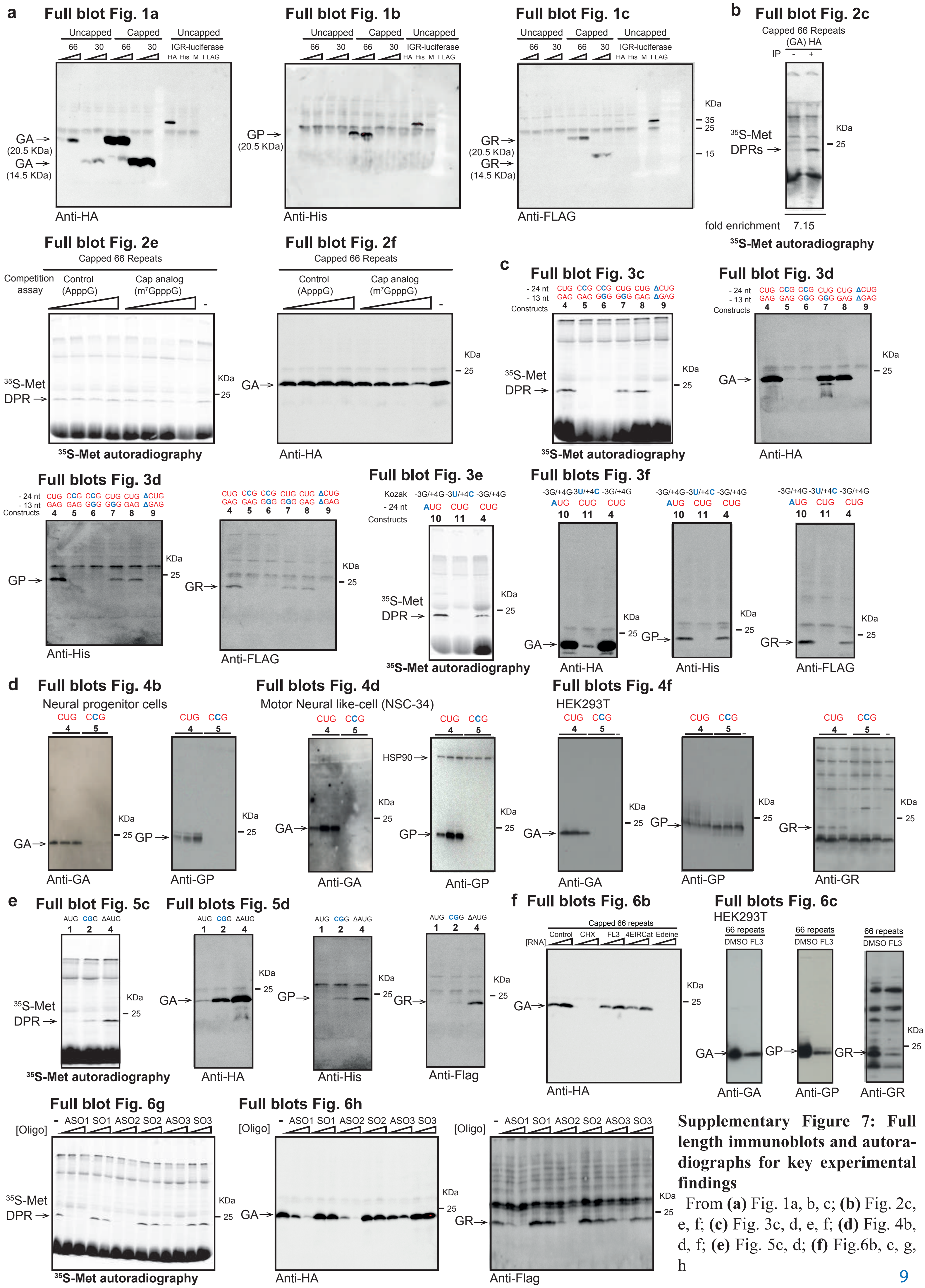


**Supplementary Figure 7: Full length immunoblots and autoradiographs for key experimental findings**

From **(a)** Fig. 1a, b, c; **(b)** Fig. 2c, e, f; **(c)** Fig. 3c, d, e, f; **(d)** Fig. 4b, d, f; **(e)** Fig. 5c, d; **(f)** Fig.6b, c, g, h

## Supplementary Table 1- Sequence of constructs

**Construct 1**

*Acgtaacctacggtgtcccgctaggaaagagaggtgcgtcaaacagcgacaagttccgcccacgtaaaag**atg**acgcttggtgtgtcagccgtccctgctgcccggttgcttctcttttgggggcggggtctagcaagagcaggtgtgggtttaggaggtgtgtgtttttgtttttcccaccctctctccccactacttgctctcacagtactcgctgagggtgaacaagaaaagacctgataaagattaaccagaagaaaacaaggagggaaacaaccgcagcctgtagcaagct**ctg**gaactcaggagtcgcgcgcta**(ggggcc)**$_{66}$ggggcgtggtcggggcgggcccgggggcgggcccggggcggggctgcggttgcggtgcctgcgcccgcggcggcggaggcgcaggcggtggcgagtggggatcaaacgcggccgc**gattacaaggacgacgacgacaag**a**tacccatacgacgttccagattacgct**agaacaaaaacttatttctgaagaagatctg**catcatcatcatcatcat**ggatcctagttaagtag

**Construct 2**

*Acgtaacctacggtgtcccgctaggaaagagaggtgcgtcaaacagcgacaagttccgcccacgtaaaag**CG**gacgcttggtgtgtcagccgtccctgctgcccggttgcttctcttttgggggcggggtctagcaagagcaggtgtgggtttaggaggtgtgtgtttttgtttttcccaccctctctccccactacttgctctcacagtactcgctgagggtgaacaagaaaagacctgataaagattaaccagaagaaaacaaggagggaaacaaccgcagcctgtagcaagct**ctg**gaactcaggagtcgcgcgcta**(ggggcc)**$_{66}$ggggcgtggtcggggcgggcccgggggcgggcccggggcggggctgcggttgcggtgcctgcgcccgcggcggcggaggcgcaggcggtggcgagtggggatcaaacgcggccgc**gattacaaggacgacgacgacaag**a**tacccatacgacgttccagattacgct**agaacaaaaacttatttctgaagaagatctg**catcatcatcatcatcat**ggatcctagttaagtag

**Construct 3**

*agtactcgctgagggtgaacaagaaaagacctgataaagattaaccagaagaaaacaaggagggaaacaaccgcagcctgtagcaagct**ctg**gaactcaggagtcgcgcgcta**(ggggcc)**$_{30}$ggggcgtggtcggggcgggcccgggggcgggcccggggcggggctgcggttgcggtgcctgcgcccgcggcggcggaggcgcaggcggtggcgagtggggatcaaacgcggccgc**gattacaaggacgacgacgacaag**a**tacccatacgacgttccagattacgct**agaacaaaaacttatttctgaagaagatctg**catcatcatcatcatcat**ggatcctagttaagtag

**Construct 4**

*agtactcgctgagggtgaacaagaaaagacctgataaagattaaccagaagaaaacaaggagggaaacaaccgcagcctgtagcaagct**ctg**gaactcaggagtcgcgcgcta**(ggggcc)**$_{66}$ggggcgtggtcggggcgggcccgggggcgggcccggggcggggctgcggttgcggtgcctgcgcccgcggcggcggaggcgcaggcggtggcgagtggggatcaaacgcggccgc**gattacaaggacgacgacgacaag**a**tacccatacgacgttccagattacgct**agaacaaaaacttatttctgaagaagatctg**catcatcatcatcatcat**ggatcctagttaagtag

**Construct 5**

*agtactcgctgagggtgaacaagaaaagacctgataaagattaaccagaagaaaacaaggagggaaacaaccgcagcctgtagcaagct**cCg**gaactcaggagtcgcgcgcta**(ggggcc)**$_{66}$ggggcgtggtcggggcgggcccgggggcgggcccggggcggggctgcggttgcggtgcctgcgcccgcggcggcggaggcgcaggcggtggcgagtggggatcaaacgcggccgc**gattacaaggacgacgacgacaag**a**tacccatacgacgttccagattacgct**agaacaaaaacttatttctgaagaagatctg**catcatcatcatcatcat**ggatcctagttaagtag

**Construct 6**

*agtactcgctgagggtgaacaagaaaagacctgataaagattaaccagaagaaaacaaggagggaaacaaccgcagcctgtagcaagct**cCg**gaactcagg**G**gtcgcgcgcta**(ggggcc)**$_{66}$ggggcgtggtcggggcgggcccgggggcgggcccggggcggggctgcggttgcggtgcctgcgcccgcggcggcggaggcgcaggcggtggcgagtggggatcaaacgcggccgc**gattacaaggacgacgacgacaag**a**tacccatacgacgttccagattacgct**agaacaaaaacttatttctgaagaagatctg**catcatcatcatcatcat**ggatcctagttaagtag

**Construct 7**

*agtactcgctgagggtgaacaagaaaagacctgataaagattaaccagaagaaaacaaggagggaaacaaccgcagcctgtagcaagct**ctg**gaactcagg**G**gtcgcgcgcta**(ggggcc)**$_{66}$ggggcgtggtcggggcgggcccgggggcgggcccggggcggggctgcggttgcggtgcctgcgcccgcggcggcggaggcgcaggcggtggcgagtggggatcaaacgcggccgc**gattacaaggacgacgacgacaag**a**tacccatacgacgttccagattacgct**agaacaaaaacttatttctgaagaagatctg**catcatcatcatcatcat**ggatcctagttaagtag

**Construct 8**

*tagcaagct**ctg**gaactcaggagtcgcgcgcta**(ggggcc)**$_{66}$ggggcgtggtcggggcgggcccgggggcgggcccggggcggggctgcggttgcggtgcctgcgcccgcggcggcggaggcgcaggcggtggcgagtggggatcaaacgcggccgc**gattacaaggacgacgacgacaag**a**tacccatacgacgttccagattacgct**agaacaaaaacttatttctgaagaagatctg**catcatcatcatcatcat**ggatcctagttaagtag

**Construct 9**

*gcgcgcta**(ggggcc)**$_{66}$ggggcgtggtcggggcgggcccgggggcgggcccggggcggggctgcggttgcggtgcctgcgcccgcggcggcggaggcgcaggcggtggcgagtggggatcaaacgcggccgc**gattacaaggacgacgacgacaag**a**tacccatacgacgttccagattacgct**agaacaaaaacttatttctgaagaagatctg**catcatcatcatcatcat**ggatcctagttaagtag

**Construct 10**

*agtactcgctgagggtgaacaagaaaagacctgataaagattaaccagaagaaaacaaggagggaaacaaccgcagcctgtagcaagct**Atg**gaactcaggagtcgcgcgcta**(ggggcc)**$_{66}$ggggcgtggtcggggcgggcccgggggcgggcccggggcggggctgcggttgcggtgcctgcgcccgcggcggcggaggcgcaggcggtggcgagtggggatcaaacgcggccgc**gattacaaggacgacgacgacaag**a**tacccatacgacgttccagattacgct**agaacaaaaacttatttctgaagaagatctg**catcatcatcatcatcat**ggatcctagttaagtag

**Construct 11**

*agtactcgctgagggtgaacaagaaaagacctgataaagattaaccagaagaaaacaaggagggaaacaaccgcagcctgtagcaa**T**ct**ctg**Caactcaggagtcgcgcgcta**(ggggcc)**$_{66}$ggggcgtggtcggggcgggcccgggggcgggcccggggcggggctgcggttgcggtgcctgcgcccgcggcggcggaggcgcaggcggtggcgagtggggatcaaacgcggccgc**gattacaaggacgacgacgacaag**a**tacccatacgacgttccagattacgct**agaacaaaaacttatttctgaagaagatctg**catcatcatcatcatcat**ggatcctagttaagtag

*Start of the human sequence

Highlighted in grey Exon 1A and Exon 1B
In orange: Start and stop codon for the uORF
In blue: near-cognate start codon for RAN translation
In red: various point mutations inserted by site-directed mutagenesis
**In green: FLAG, HA and His tags**

**Supplementary Table 2. Oligonucleotides used for the generation of C9ORF72 constructs**

| Mutation | Forward (5'-3') | Reverse (5'-3') |
|---|---|---|
| Construct #**1**. Full sequence (320bp) upstream of the $(G_4C_2)_{66}$ repeats with T7 Promoter | *Primer 1*:<br>agctttaatacgactcactataggacgtaacctacggtgtcccgctaggaaagagaggtgcgtcaaacagcgacaagttccgcccacgtaaaagatgacgcttggtgtgtcagccgtccctgctgcccggttgcttctcttttgggggcggggtctagcaagagcaggtgtgggtttaggag<br>*Primer 2*:<br>gtgtgtgtttttgtttttcccaccctctctccccactacttgctctcacagtactcgctgagggtgaacaagaaaagacctgataaagattaaccagaagaaaacaaggagggaaacaaccgcagcctgtagcaagctctggaactcaggagtcg | *Primer 1*:<br>cacacacctcctaaacccacacctgctcttgctagaccccgcccccaaaagagaagcaaccgggcagcagggacggctgacacaccaagcgtcatcttttacgtgggcggaacttgtcgctgtttgacgcacctctctttcctagcgggacaccgtaggttacgtcctatagtgagtcgtattaa<br>*Primer 2:*<br>cgcgcgactcctgagttccagagcttgctacaggctgcggttgtttccctccttgttttcttctggttaatctttatcaggtcttttcttgttcaccctcagcgagtactgtgagagcaagtagtggggagagagggtgggaaaaacaaaaa |
| Construct #**2**. Full sequence (320bp) upstream of $(G_4C_2)_{66}$ repeats with AUG>**CG**G mutation and T7 Promoter | *Primer 1*:<br>agctttaatacgactcactataggacgtaacctacggtgtcccgctaggaaagagaggtgcgtcaaacagcgacaagttccgcccacgtaaaag**CG**gacgcttggtgtgtcagccgtccctgctgcccggttgcttctcttttgggggcggggtctagcaagagcaggtgtgggtttaggag<br>*Primer 2*:<br>gtgtgtgtttttgtttttcccaccctctctccccactacttgctctcacagtactcgctgagggtgaacaagaaaagacctgataaagattaaccagaagaaaacaaggagggaaacaaccgcagcctgtagcaagctctggaactcaggagtcg | *Primer 1*:<br>cacacacctcctaaacccacacctgctcttgctagaccccgcccccaaaagagaagcaaccgggcagcagggacggctgacacaccaagcgtc**CG**cttttacgtgggcggaacttgtcgctgtttgacgcacctctctttcctagcgggacaccgtaggttacgtcctatagtgagtcgtattaa<br>*Primer 2:*<br>cgcgcgactcctgagttccagagcttgctacaggctgcggttgtttccctccttgttttcttctggttaatctttatcaggtcttttcttgttcaccctcagcgagtactgtgagagcaagtagtggggagagagggtgggaaaaacaaaaa |
| Constructs #**3** and #**4**. 113 bp upstream of the $(G_4C_2)_{30}$ or $(G_4C_2)_{66}$ repeats, with T7 promoter | agcttctcgagtaatacgactcactatagga | agcttcctatagatgagtcgtattactcgaga |
| Construct #**5**. 113 bp upstream of the $(G_4C_2)_{66}$ repeats with CUG>C**C**G mutation and T7 promoter | agctttaatacgactcactataggagtactcgctgagggtgaacaagaaaagacctgataaagattaaccagaagaaaacaaggagggaaacaaccgcagcctgtagcaagctc**C**ggaactcaggagtcgcgcgcggtaccgagct | cggtaccgcgcgcgactcctgagttcc**G**gagcttgctacaggctgcggttgtttccctccttgttttcttctggttaatctttatcaggtcttttcttgttcaccctcagcgagtactcctatagtgagtcgtattaa |
| Construct #**6**. 113 bp upstream of the $(G_4C_2)_{66}$ repeats with GAG>G**G**G mutation and T7 promoter | agctttaatacgactcactataggagtactcgctgagggtgaacaagaaaagacctgataaagattaaccagaagaaaacaaggagggaaacaaccgcagcctgtagcaagctctggaactcagg**G**gtcgcgcgcggtaccgagct | cggtaccgcgcgcgac**C**cctgagttccagagcttgctacaggctgcggttgtttccctccttgttttcttctggttaatctttatcaggtcttttcttgttcaccctcagcgagtactcctatagtgagtcgtattaa |
| Construct #**7**. 113 bp upstream of the $(G_4C_2)_{66}$ repeats with GAG>G**G**G + CUG>C**C**G mutations and T7 promoter | agctttaatacgactcactataggagtactcgctgagggtgaacaagaaaagacctgataaagattaaccagaagaaaacaaggagggaaacaaccgcagcctgtagcaagctc**C**ggaactcagg**G**gtcgcgcgcggtaccgagct | cggtaccgcgcgcgac**C**cctgagttcc**G**gagcttgctacaggctgcggttgtttccctccttgttttcttctggttaatctttatcaggtcttttcttgttcaccctcagcgagtactcctatagtgagtcgtattaa |
| Construct #**8**. 33 bp upstream of the $(G_4C_2)_{66}$ repeats with T7 promoter | agctttaatacgactcactataggtagcaagctctggaactcaggagtcg | cgcgcgactcctgagttccagagcttgctacctatagtgagtcgtattaa |
| Construct #**9**. 8 bp upstream of the $(G_4C_2)_{66}$ repeats with T7 promoter | agctttaatacgactcactatagggg | cgcgccctatagtgagtcgtattaa |
| Construct #**10**. 113 bp | agctttaatacgactcactataggagtactcgctgagg | cggtaccgcgcgcgactcctgagttcca**T**gagcttgcta |

| | | |
|---|---|---|
| upstream of the $(G_4C_2)_{66}$ repeats with CTG>ATG, with T7 promoter | gtgaacaagaaaagacctgataaagattaaccagaa gaaaacaaggagggaaacaaccgcagcctgtagca agctcAtggaactcaggagtcgcgcgcggtaccga gct | caggctgcggttgtttccctccttgttttcttctggttaatcttt atcaggtcttttcttgttcaccctcagcgagtactcctatagt gagtcgtattaa |
| Plasmid **#11**. 113 bp of 5' $(G_4C_2)_{repeats}$ flanking sequence with GCTCTGG>TCTCTGC and T7 promoter | agctttaatacgactcactataggagtactcgctgagg gtgaacaagaaaagacctgataaagattaaccagaa gaaaacaaggagggaaacaaccgcagcctgtagca aTctctgCaactcaggagtcgcgcgcggtaccgag ct | cggtaccgcgcgcgactcctgagttGcagagTtgcta caggctgcggttgtttccctccttgttttcttctggttaatcttt atcaggtcttttcttgttcaccctcagcgagtactcctatagt gagtcgtattaa |
| Plasmid **#12**. Full sequence of CrPV –IGR upstream of Renilla luciferase coding sequence | *T7-CrPV2* caacaaatattaatacgactcactataggcaaaaatgt gatcttgcttg | *Primer 1*: Rev Renilla-HA gaattatgcataatccggcacatcatacggatattgttcattt ttgaggactcgctcg *Primer 2*: Rev Renilla-His gaattagtggtggtggtggtggtgttgttcatttttgaggact cgctcg *Primer 3*: Rev Renilla-FLAG gaattacttgtcgtcgtcgtccttgtaatcttgttgttcattttg aggactcgctcg |
| Plasmid **#13**. Full sequence of 5' β-globin 5'UTR upstream of Renilla luciferase coding sequence | *T7-β-globin-5'UTR* caacaaatattaatacgactcactataggacatttgctt ctgacacaactgtg | ccggttattgttcatttttgagaactcgctc |

**Supplementary Table 3. RNA antisense oligonucleotides targeting regions upstream of the C9ORF72 $G_4C_2$ transcripts**

| ID number | Target | Sequence (5'>3') |
|---|---|---|
| RNA-SO1, Fig. 6 f,g | Control | gcugagggugaacaagaaaagacc |
| RNA-ASO1, Fig. 6 f,g | Region 5' of the $G_4C_2$ transcripts | ggucuuuucuuguucacccucagc |
| RNA-SO2, Fig. 6 f,g | Control | gaagaaaacaaggagggaaacaacc |
| RNA-ASO2, Fig. 6 f,g | Region 5' of the $G_4C_2$ transcripts | gguuguuucccuccuuguuuucuuc |
| RNA-SO3, Fig. 6 f,g | Control | gccuguagcaagcucuggaacucaggaguc |
| RNA-ASO3, Fig. 6 f,g | Region 5' of the $G_4C_2$ transcripts | gacuccugaguuccagagcuugcuacaggc |

**Supplementary Table 4. Antibodies used for immuno-blot, -precipitation, and -histochemistry**

| Antibody | Application | Species | Dilution | Reference |
|---|---|---|---|---|
| Poly-Gly-Ala (GA) | WB, IHC | Rabbit | 1:1000 | Rb 4334 *Jiang et al., 2016* |
| Anti-Gly-Pro (GP) | WB, IHC | Rabbit | 1:1000 | Rb 4335 *Jiang et al., 2016* |
| Anti-Gly-Arg (GR) | WB, IHC | Rabbit | 1:1000 | Rb 4995 *Jiang et al., 2016* |
| Flag | WB, IP | Mouse | 1:1000 | Sigma F1804 |
| GFP | WB | Rabbit | 1:1000 | Abcam #290 |
| HA | WB, IP | Mouse | 1:500 | Santa Cruz #7392 |
| HA | WB | Rat | 1:1000 | Sigma #3F10 |
| $His_{5X}$ | WB, IP | Mouse | 1:400 | Qiagen #34660 |
| Hsp90 | WB | Rabbit | 1:2000 | Stressgen ADI-SPA-846-D |

WB: immunoblot, IHC: immunohistochemistry, IP: immunoprecipitation
